# Antitumor Drug Combretastatin-A4 Phosphate Aggravates the Symptoms of Dextran Sulfate Sodium-Induced Ulcerative Colitis in Mice

**DOI:** 10.3389/fphar.2020.00339

**Published:** 2020-03-24

**Authors:** Zhengshan Tang, Dehui Xiong, Jianhui Song, Mao Ye, Jing Liu, Zi Wang, Lei Zhang, Xiaojuan Xiao

**Affiliations:** ^1^Hepatobiliary and Enteric Surgery Research Center, Xiangya Hospital, Central South University, Changsha, China; ^2^School of Life Sciences & Hunan Province Key Laboratory of Basic and Applied Hematology, Central South University, Changsha, China; ^3^Molecular Science and Biomedicine Laboratory, State Key Laboratory for Chemo/Biosensing and Chemometrics, College of Biology, College of Chemistry and Chemical Engineering, Collaborative Innovation Center for Molecular Engineering for Theranostics, Hunan University, Changsha, China; ^4^Department of Nephrology, The Second Xiangya Hospital of Central South University, Hunan Key Laboratory of Kidney Disease and Blood Purification, Changsha, China

**Keywords:** ulcerative colitis, combretastatin-A4 phosphate, dextran sodium sulfate, proinflammatory cytokines, inflammatory cells

## Abstract

Ulcerative colitis (UC) is an idiopathic inflammatory bowel disease (IBD) that causes long-lasting inflammation and ulcers in the innermost lining of the colon and rectum. Previous studies demonstrated that resveratrol suppresses colitis and colon cancer associated with colitis by improving glucose metabolism, but resveratrol use is limited by its low oral bioavailability. Combretastatin-A4 phosphate (CA4P) is a vascular-disrupting agent with antitumor activity. CA4P is structurally similar to resveratrol, but whether CA4P has the same effect as resveratrol on UC is not clear. In this study, we examined the pharmacological effects of CA4P administration on dextran sulfate sodium (DSS)-induced inflammation in a mouse model of UC. C57BL/6 mice were administered 2.5% DSS in the drinking water to induce acute UC. CA4P (11 mg/kg/d) was injected intraperitoneally daily. The Disease Activity Index (DAI) score and histological score were evaluated to determine the severity of UC. Colon tissues and blood samples were collected for histological analyses. The results show that CA4P plus DSS significantly decreased colon length (*P* < 0.05 versus DSS+PBS group) and body weight (*P* < 0.001 versus PBS group), while increased spleen weight (*P* < 0.01 versus DSS+PBS group), DAI score (*P* < 0.01 versus DSS+PBS group), and histological score (*P* < 0.01 versus DSS+PBS group). Moreover, CA4P exacerbated the pathological features of colitis and significantly increased proinflammatory cytokines (IL-1β, IL-6, TNF-α) and inflammatory cells (neutrophil, lymphocyte, monocyte). These findings reveal that CA4P aggravates the symptoms of DSS-induced UC and provide a key reference for the potential of CA4P as an anticancer drug.

## Introduction

Ulcerative colitis (UC) is an idiopathic inflammatory bowel disease (IBD) that causes long-lasting inflammation and sores (ulcers) in the innermost lining of large intestine (colon) and rectum (Deuring et al., 2013). UC is associated with an architectural distortion of the intestinal epithelium and impaired mucosal barrier function (Chen et al., 2014; Zhang et al., 2017a). Patients with UC generally exhibit undesirable symptoms, such as fatigue, diarrhea, and rectal bleeding (blood in the stool) (Danese et al., 2014). Disease recurrences may affect the innermost layer of the large intestine and rectum, increasing the prevalence of colorectal cancer and the risk of extraintestinal diseases, such as iritis/uveitis, arthritis, major sclerosing cholangitis, and ankylosing spondylitis (Vavricka et al., 2015; Harbord et al., 2016).

Activated dendritic cells and macrophages secrete several cytokines that actively regulate inflammation in UC (Sanchez-Munoz et al., 2008). The colonic mucosa of UC patients have increased levels of inflammatory cytokines, such as tumor necrosis factor (TNF)-α, interleukin (IL)-1β, and IL-6 (Bouguen et al., 2011). The transcription factor nuclear factor-kappa B (NF-κB) is responsible for the transactivation of these inflammatory cytokines, which results in the perpetuation of mucosal inflammation and promotes the inflammatory process in UC patients (Bonizzi and Karin, 2004; Atreya et al., 2008; Chen et al., 2018).

Combretastatin-A4 (CA4) is a natural cis-stilbene that was originally separated from *Combretumcaffrum* of South Africa (Tarade et al., 2017). It has dramatic effects on the three-dimensional shape of newly formed endothelial cells, with little or no effect on quiescent endothelial cells (Galbraith et al., 2001; Siemann et al., 2009). Since CA4 exhibits limited water solubility, water-soluble CA4P is developed as a prodrug that is rapidly dephosphorylated to the active CA4 compound *in vivo*. Previous studies have shown that resveratrol suppresses colitis and colon cancer associated with colitis *via* improved glucose metabolism (Zhang et al., 2008). Similarly, the grape seed extract (GSE), a polyphenol-based compound similar to resveratrol, decreased the severity of selected markers of DSS-induced colitis in the distal ileum and proximal colon, suggesting the potential as an adjuvant therapy for the treatment of UC (Cheah et al., 2013). However, clinical applications of resveratrol is limited by its low oral bioavailability (Shi et al., 2017). Given that CA4P is a structural analog of resveratrol, we explored whether CA4P has the same effects as resveratrol on UC. However, we found that CA4P aggravated the symptoms of DSS-induced UC. Therefore, our results suggest that CA4P may not be well suited for UC treatment and possessed potential side effects as an anticancer drug.

## Materials and Methods

### Animals

Five-week-old C57BL/6 male mice (Silaike Jingda Laboratory Animal Co Ltd, Hunan, China) were housed and fed under standard conditions at an animal care facility. Mice were maintained in a temperature- and light-controlled facility with free access to standard rodent chow and water. Experiments were performed after a 1-week acclimatization period. Mice were housed in cages with a room temperature of 25 ± 2°C and a daily 12-h light-dark cycle.

### Ulcerative Colitis Model

Five-week-old C57BL/6 male mice were weighed and randomly assigned to four groups: group A: H_2_O+PBS (control, n=16); group B: H_2_O+CA4P (n=16); group C: DSS+PBS (n=16); and group D: DSS+CA4P (n=16). Group A and B were administered the drinking water for 0–9 days. Group C and D were administered 2.5% DSS in the drinking water for 0–9 days to induce acute UC. Phosphate-buffered saline (PBS) or 11 mg/kg/d CA4P (quality standard: ≥98%; molecular formula: C_18_H_19_Na_2_O_8_P, MW: 440.30; Meilun, China) were administered by intraperitoneal injection for 0–9 days. All drugs were freshly prepared on the experiment day. Body weight, food intake, stool consistency, and the presence of gross bleeding were assessed daily. Mice were euthanized *via* exsanguination under general anesthesia with spontaneous inhalation of isoflurane at the ending day (9 day). Blood samples were collected from the inferior vena cava. Several tissues, including the intestines and/or spleen, were excised. The colon from the ileocecal junction to the anus was excised and cut open lengthwise or into small pieces for use in further experiments.

### Disease Activity Index

Severity of colitis was evaluated by DAI based on the previously described scoring system (Alex et al., 2009). DAI is the summation of the weight loss index (0–4); stool consistency index (0–4); and rectal bleeding index (0–4). DAI = (combined score of weight loss, stool consistency and bleeding)/3. Scores were assessed as follows: for weight loss, a score of 0 for body weight within the 1% of baseline; 1 for a 1–5% loss; 2 for a 6–10% loss; 3 for an 11–15% loss; and 4 for weight losses over 15%. For stool consistency, a value of 0 was assigned for well-formed pellets, 2 for loose stool, and 4 for diarrhea. Rectal bleeding was graded 0 for negative, 2 for fecal occult blood test positive, and 4 for gross bleeding (**Table 1**). Occult blood in feces was detected by using benzidine method.

**Table 1:** Criteria for DAI scoring^(a)^.

Score	Weight loss (%)	Stool consistency^(b)^	Bleeding
0	None	Normal	No bleeding
1	1-5	–	–
2	6-10	Loose stoold	Slight bleding
3	11-15	–	–
4	>15	Watery diarrhea	Gross bleeding

^(a)^Disease Activity Index = (combined score of weight loss, stool constancy, and bleeding)/3. ^(b)^ Normal stool = well-formed pellets; loose stool = pasty stool that did not stick to the anus; and diarrhea = liquid stool that stuck to the anus.

### Histopathological Analysis

Mice were sacrificed at the end of the experiment. The colon was dissected and flushed with ice-cold PBS. Samples were fixed in 10% neutral-buffered formalin for 2 days at 4°C, embedded in paraffin, and cut in sequential longitudinal sections of 5-mm thickness using a sliding microtome (Leica SM2010R, Leica Microsystems, Wetzler, Germany). Tissue sections were deparaffinized using xylene, dehydrated in a gradient of alcohol solutions, and stained with hematoxylin and eosin (H&E) as described previously (Hu et al., 2016). Severity of colitis was evaluated in sections stained with H&E by 15 randomly selected fields were inspected in each section by two independent observers blinded to the experimental conditions according to the modified criteria of Hamamoto et al. (Hamamoto et al., 1999), and assigned scores as follows: grade 0, normal colonic mucosa; grade 1, loss of one-third of the crypts; grade 2, loss of two-thirds of the crypts; grade 3, lamina propria covered with a single layer of epithelial cells with mild inflammatory cell infiltration; and grade 4, erosions and marked inflammatory cell infiltration. After grading the 15 fields, the mean grade was calculated for each section and expressed as histological score.

Immunohistochemical analyses of TNF-α (Abcam, ab1793) expression in colon tissue were performed in 5-µm-thick tissue sections. Sections were deparaffinized using xylene, dehydrated in a gradient of alcohol solutions, incubated in 3% H_2_O_2_-methanol for 20 min at room temperature to quench endogenous peroxidase activity and incubated with primary antibodies (diluted 1:150) against proteins of interest for 1 h. Biotin-labeled goat anti-mouse IgG antibodies (Abcam, ab6788) were used at 1:200 dilution. Slides were stained using liquid diaminobenzidine tetrahydrochloride (DAB+), which is a high-sensitivity substrate-chromogen system. Slides were visualized under an Olympus BX40 light microscope.

As described previously (Johansson et al., 2001; Wang et al., 2016; Sun et al., 2019), the positive expression of TNF-α was evaluated by the cytoplasmic staining intensity using an image pro plus software (version 6.0). The mean value of TNF-α positive cell numbers in one sample side was calculated as the average of positive cell numbers divided by total cell numbers from five random fields.

### Western Blotting Assay

As described previously (Wang et al., 2017), the freshly colonic tissues were excised and fragmented using a scalpel. 500 ug–1 mg tissue portions were lysed for 30 min on ice with RIPA lysis buffer (Beyotime) in the presence of cocktail protease inhibitor (Roche) and phoSTOP phosphatase inhibitor (Roche). An additional step of sonication was also performed. Protein extracts (35 µg) were boiled and subjected to 10% SDS-PAGE gel before transfer to nitrocellulose membranes. The membranes were blocked for 2 h in PBST (PBS with 0.5% Tween 20) with 5% non-fat dry milk (Bio-Rad) and incubated with specific primary antibodies at 4°C overnight. Appropriate HRP-conjugated second antibodies (Cell Signaling Technology, #7076) were used at 1:3,000 dilution for 2 h at room temperature. Signals were detected by ECL HRP substrate (Advansta). The following primary antibodies were used: mouseβ-actin (Santa Cruz, sc-47778), mouse NF-κB p65 (Cell Signaling Technology, #6956), and mouse TNF-α (Abcam, ab1793).

### Quantitative Real Time Polymerase Chain Reaction (QRT-PCR)

RNA was extracted from excised colons stored at −80°C using an RNA extraction kit (Feiluomaige, Beijing, China) according to the manufacturer’s instructions. First-strand cDNA was produced using TaKaRaTaq™ (Takara) and KOD FX (TOYOBO, Osaka, Japan) according to the manufacturers’ instructions in a PCR instrument (Mastercycler, Eppendorf). The expression levels of target genes were determined using the SYBR^®^Premix Ex Taq™ kit (Takara). The following cycling conditions were used: 95°C for 3 min, followed by 45 cycles of 95°C for 30 s, 58°C for 30 s, and elongation at 72°C for 30 s. All gene primers were synthesized at Qinke Biotech. **Table 2** lists the target-specific QRT-PCR primers designed for IL-1β, IL-6, TNF-α, and β-actin.

**Table 2:** QRT-PCR primers used in this study.

Gene		Sequence (5–3′)
IL-1β	Forward	GCAACTGTTCCTGAACTCAACT
	Reverse	ATCTTTTGGGGTCCGTCAACT
TNF-α	Forward	CACGCTCTTCTGTCTACTGAA
	Reverse	GGCTACAGGCTTGTCACTCGA
IL-6	Forward	GAGGATACCACTCCCAACAGACC
	Reverse	AAGTGCATCATCGTTGTTCATACA
β-Actin	Forward	GCTCTGGCTCCTAGCACCAT
	Reverse	GCCACCGATCCACACAGAGT

### Ethics

The Animal Care and Experiment Committee of Hunan University approved the protocol for animal experiments. The investigation conformed to the Guide for the Care and Use of Laboratory Animals published by the US National Institutes of Health (NIH Publication No. 85-23, revised 1996).

### Statistical Analyses

All statistical analyses were performed with the SPSS 16.0 statistical software package. Student’s t-test was used to determine the significance of the differences between the control and the experimental groups. Error bars were used to indicate the standard deviation of the data, and *P* < 0.05 was considered statistically significant.

## Results

### CA4P Significantly Reduced the Body Weight of DSS-Treated C57BL/6 Mice and Increased the DAI

**Figure 1:**
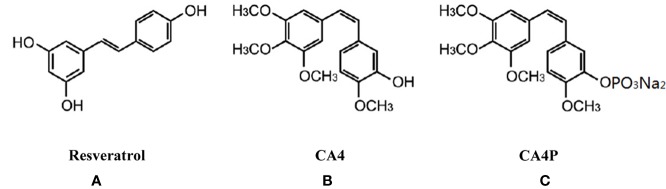
Chemical structure of resveratrol **(A)**, combretastatin-A4 (CA4) **(B)**, and combretastatin-A4 phosphate (CA4P) **(C)**. CA4, combretastatin-A4; CA4P, combretastatin-A4 phosphate.

**Figure 2:**
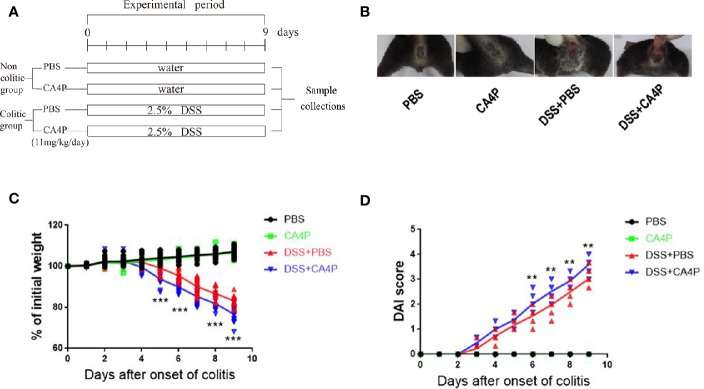
The effects of CA4P on the DAI and body weight of DSS-treated C57BL/6 mice. Ulcerative colitis was induced *via* the administration of 2.5% DSS for 9 days. **(A)** Experimental process of inducing UC using 2.5% DSS. **(B)** Rectal bleeding was observed in each group on the 9^th^ day. **(C)** Body weight changes and **(D)** DAI scores were evaluated daily. Values represent the mean ± SD (n = 16 for each group). **P < 0.01, ***P < 0.001 versus PBS group based on the Student’s t-test. CA4P, combretastatin-A4 phosphate; DAI, Disease Activity Index; DSS, dextran sulfate sodium; UC, ulcerative colitis.

DSS-induced mouse colitis is a well-established preclinical model that exhibits many phenotypic features of human UC (Low et al., 2013). Patients with UC often exhibit weight loss and an increased DAI. Given that CA4P and resveratrol are structurally similar (**Figures 1A–C**), we first examined the effect of CA4P on DSS-induced UC mice in accordance with the experimental design scheme (**Figure 2A**). Severe clinical symptoms, including rectal bleeding, were observed on day 9 in DSS-treated mice. Rectal bleeding was generally more severe in the DSS+CA4P group than in the DSS+PBS group (**Figure 2B**). Mice with DSS-induced UC exhibited 13% body weight loss compared to mice in the CA4P and PBS groups, whereas DSS-induced UC mice treated with 11 mg/kg/d CA4P exhibited 9% body weight loss compared to mice in the DSS+PBS group (**Figure 2C**). The DAI scores of mice in the DSS-treated group were significantly higher than those of mice in the CA4P and PBS groups, and mice in the DSS+CA4P group had further increased DAI scores (**Figure 2D**). These results demonstrated that CA4P significantly reduced bodyweight and increased the DAI in DSS-treated C57BL/6 mice.

### CA4P Significantly Reduced the Colorectal Length and Spleen Weight of DSS-Treated C57BL/6 Mice

**Figure 3:**
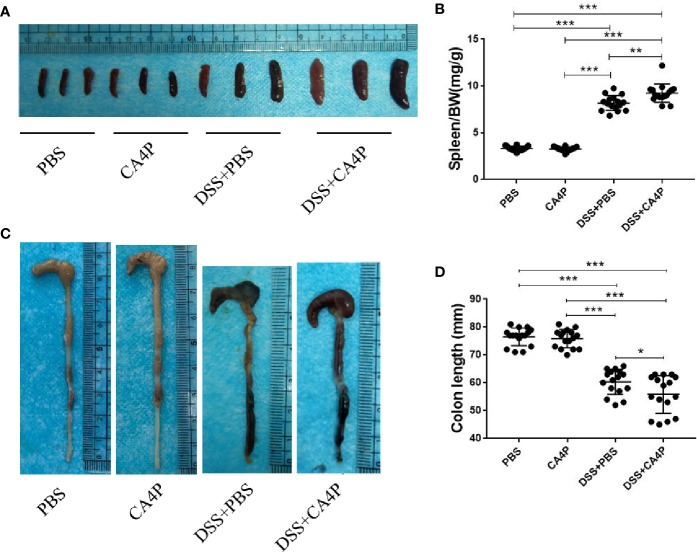
Colon length and spleen weight in mice with DSS-induced colitis. **(A)** Spleen weight increased in mice receiving DSS. Spleens were harvested on day 9, and spleen weights are shown. **(B)** Average spleen weight (mg)/body weight (g) ratio per group are shown. **(C)** Colons were removed from DSS-treated mice on day 9, opened longitudinally, washed with PBS, and then photographed. **(D)** The length of each colon was measured. Data are presented as medians (n= 16 for each group). Values represent the mean ± SD (n = 16 for each group). *P < 0.05, ***P < 0.001 based on the Student’s t-test. DSS, dextran sulfate sodium.

Colorectal shortening and spleen enlargement reflect the extent of colon damage during acute DSS-induced colitis (Hendrickson et al., 2002). We found that spleen weight increased in DSS-treated mice, and this increase was more significant in the DSS+CA4P group than in the DSS+PBS group (**Figure 3A**). Meanwhile, the spleen/body weight ratio was significantly elevated in the DSS+PBS and DSS+CA4P groups. There was no significant difference between the CA4P and PBS groups (**Figure 3B**). Moreover, the average colon length was approximately 82 mm in the PBS group and 81 mm in the CA4P group. While it decreased to 68 mm in the DSS+PBS group and to 62 mm in the DSS+CA4P group (**Figure 3C**). The colon was significantly shorter in the DSS+CA4P group than in the DSS+PBS group, but there was no significant difference in the colon length between the CA4P and PBS groups (**Figure 3D**). These results suggest that CA4P treatment exacerbated DSS-induced acute colitis.

### Histological Analysis to Assess the Therapeutic Effects of CA4P on UC

**Figure 4:**
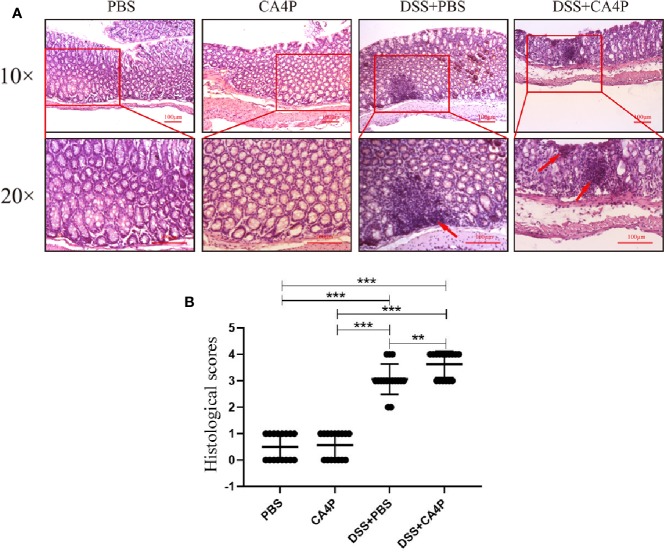
The effects of CA4P on colonic histology. **(A)** Histological changes in colonic tissue were determined using H&E staining and light microscopy (×10 and ×20). Arrows indicate basal lymphoid aggregates. **(B)** The microscopic sections were studied for histological evaluation and scoring. Values represent the mean ± SD (n = 16 for each group). **P < 0.01, ***P < 0.001 based on the Student’s t-test. CA4P, combretastatin-A4 phosphate; H&E, hematoxylin and eosin.

Colonic inflammation and mucosal damage were assessed *via* pathological examination of the colon after H&E staining. Representative results are shown in **Figure 4A**. Colon tissue sections from mice in the PBS and CA4P groups exhibited intact surface epithelium, stroma, cryptal glands, and submucosa. However, colon sections from the two groups of DSS-treated mice exhibited cryptitis, branched crypts, inflammatory cell infiltration, decreased numbers of goblet cells and crypts, and extensive submucosal edema (**Figure 4A**). Moreover, the intestinal mucosal structure of the DSS+CA4P group was more disordered than that of the DSS+PBS group. At the same time, inflammatory cell infiltration and histological damage were more serious (**Figure 4A**). Specifically, compared to DSS group, more cryptitis (neutrophilic infiltration of crypts) and basal lymphoid aggregates (prominent nodular lymphocyte aggregates located at the border of the submucosa) were observed in DSS+CA4P group (Where the arrow points at in **Figure 4A**). Correspondingly, mice treated with DSS+PBS or DSS+CA4P had significantly elevated histological scores compared to the PBS and CA4P groups. The histological score was approximately 15% higher in the DSS+CA4P group than in the DSS+PBS group (**Figure 4B**).

### CA4P Significantly Increased the Expression of Inflammatory Factors and Levels of Inflammatory Cells in DSS-Treated C57BL/6 Mice

**Figure 5:**
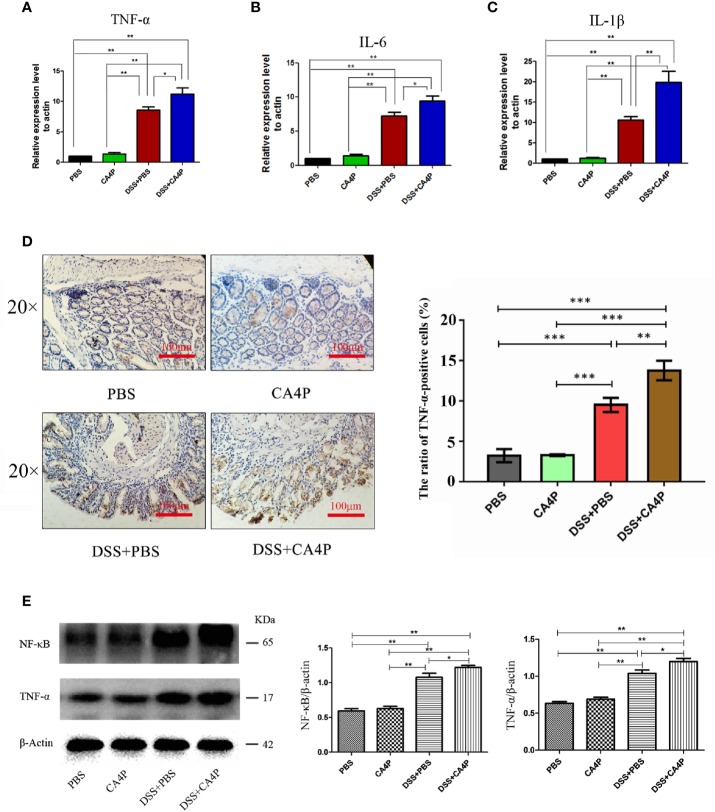
The effects of CA4P on TNF-α, IL-6, and IL-1β expression in colons from mice with DSS-induced UC. The relative mRNA expression of **(A)** TNF-α, **(B)** IL-6, and **(C)** IL-1β was measured. Data are presented as the mean ± SD (n = 8 for each group). *P < 0.05, **P < 0.01 based on the Student’s t-test. **(D)** Representative images of immunohistochemical staining of TNF-α in colonic tissues (×40, scale bar=100 μm). The brown color represents positive staining for TNF-α expression. Cell nuclei were counterstained with hematoxylin (blue). Right panel: The cell ratios are presented as the mean value of TNF-α positive cell numbers ± SD (n = 3 sample sides for each group). **(E)** Western blotting analysis of nuclear factor-kappa B (NF-κB) and TNF-α in four group of colonic tissues. β-actin was used as a loading control. Right panel: densitometric analysis (protein quantification) of western blotting signals using image J software. Fold change in each protein level normalized to β-actin is shown numerically. The data are the mean ± SD of three independent experiments, *P <0.05, **P < 0.01, ***P < 0.001 based on the Student’s t-test. CA4P, combretastatin-A4 phosphate; DSS, dextran sulfate sodium; NF-κB, nuclear factor-kappa B; TNF, tumor necrosis factor; IL, interleukin; UC, ulcerative colitis.

A key feature of DSS-induced colitis is the increased secretion of proinflammatory cytokines (Zhang et al., 2017b). Large numbers of infiltrates appear in inflammatory lesions in DSS-induced acute colitis, primarily consisting of T and B lymphocytes, macrophages, and neutrophils, which produce various proinflammatory cytokines such as TNF-α, IL-6, and IL-1β (Garside, 1999; Egger et al., 2000). We found that the relative mRNA expression of TNF-α, IL-6, and IL-1β in the colonic tissue of DSS-induced UC mice was increased significantly (**Figures 5A–C**). The relative mRNA expression of TNF-α, IL-6, and IL-1β in the colon tissue was significantly higher in the DSS+CA4P group than in the DSS-induced UC model group (**Figures 5A–C**).

**Figure 6:**
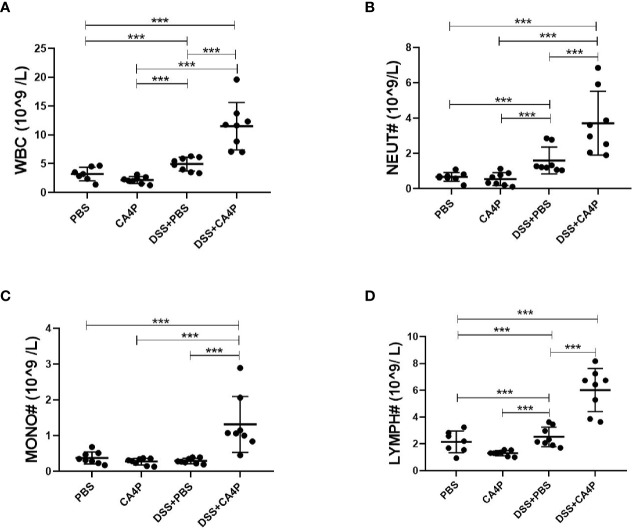
The effects of CA4P on peripheral blood leukocytes from mice with DSS-induced UC. Peripheral blood was collected from mice with four different kinds of treatment as indicated in the figure (n = 7 for the PBS group, n=8 for other groups). Peripheral blood cell classification, including white blood cells/WBC **(A)**, neutrophils/NEUT **(B)**, monocytes/MONO **(C)**, and lymphocytes/LYMPH **(D)**, and cell counting using an automatic blood cell analyzer. Data are presented as the mean ± SD. ***P < 0.001 based on the Student’s t-test. CA4P, combretastatin-A4 phosphate; DSS, dextran sulfate sodium; UC, ulcerative colitis.

Immunohistochemical analysis revealed that the number of TNF-α-positive cells was higher in DSS-treated mouse colonic mucosa than in corresponding control mucosa (**Figure 5D**). The protein expression of TNF-α and upstream transcription factor NF-κB that is required for maximal transcription of many cytokines, including TNF-α, IL-6, and IL-1β (Blackwell and Christman, 1997), were higher in the DSS+CA4P group than in the DSS group (**Figure 5E**). In order to explore which kind of inflammatory cells are modulated by DSS+CA4P. We conducted both peripheral blood cell classification and counting using an automatic blood cell analyzer (Sysmex XT-2000i, Sysmex Corporation, Kobe, Japan). We found that CA4P plus DSS treatment could significantly increase the levels of white blood cells, especially for neutrophil, lymphocyte, and monocyte (**Figures 6A–D**). These cells are key inflammatory cells that secrete proinflammatory cytokines, including TNF-α (secreted by neutrophil, lymphocyte, monocyte), IL-6 (neutrophil, lymphocyte, monocyte), and IL-1β (neutrophil, lymphocyte, monocyte) (Wirtz et al., 2004; Tecchio et al., 2014; Hadadi et al., 2016; Rizavi et al., 2016). Collectively, these results suggest that CA4P aggravates DSS-induced colitis in mice via promoting inflammatory cell levels and infiltration, as well as release of proinflammatory cytokines

## Discussion

Relevant studies have shown that resveratrol can effectively suppress colitis and colon cancer, and alleviate diabetic conditions by improving sugar metabolism. However, resveratrol has low oral absorption and utilization, which limits its clinical application. CA4, a resveratrol analog, also alleviates diabetic conditions by improving glucose metabolism. CA4P, a water-soluble prodrug of CA4, is used as a vascular-disrupting agent for the treatment of ovarian, glioma, non-small cell lung cancer (NSCLC), pancreatic neuroendocrine tumors, and etc. (Grisham et al., 2018). CA4P monotherapy is well tolerated, and most adverse events during cancer treatment are mild to moderate in intensity. To data, whether CA4P is able to treat colitis is not clear. In this study, to clarify whether CA4P has the functional activity in treating colitis. Healthy C57BL/6 mice were treated with 2.5% DSS for consecutive 9 days to establish a mouse UC model. The results showed that mice treated with DSS developed symptoms such as diarrhea, bloody stools, weight loss, and poor drinking water. Moreover, the DSS-treated mice had a much higher DAI than the untreated mice, the length of the colorectum became shorter, and the weight of the spleen became larger, indicating that the UC model was successfully established.

The experimental results showed that spleen weight and volume were significantly increased and the length of the colorectum was significantly shortened in the CA4P+DSS treatment group, compared to the PBS or DSS+PBS group. Moreover, inflammatory cell infiltration observed in the pathological section of the colorectal tissue was more severe in CA4P+DSS treatment group than that in other three groups. However, mice in the CA4P group did not show symptoms of the disease, and there was no significant difference in the performance between PBS group and CA4P group. These results suggested that CA4P does not affect the physiological activity of normal mice. At the same time, CA4P does not play a therapeutic remission effect on UC model mice, but aggravates the disease symptoms of UC model mice.

Both CA4P and resveratrol belong to stilbenes, 1,2-diphenylethen derivatives. Although they are structurally similar, the former belongs to cis-stilbenes, while the latter belongs to trans-stilbenes, which determines they have different biochemical properties (Zhao et al., 2018). The reason why they don’t play the same role in colitis may be due to different effects on inflammation. For resveratrol, Samsami-kor et al found that resveratrol improved quality of life and disease clinical colitis activity at least partially through inflammation reduction in patients with UC. Resveratrol led to a significant reduction in serum level of inflammatory factors and the activity of NF-κB and TNF-α in peripheral blood mononuclear cells (PBMCs) (Samsami-Kor et al., 2015). Similarly, Cui et al demonstrated that resveratrol mixed in food ameliorates DSS-induced colitis in mice. Resveratrol significantly improves inflammation score, down regulates the percentage of neutrophils in the mesenteric lymph nodes and lamina propiria, and modulates CD3+ T cells that express tumor necrosis factor-alpha and interferon gamma (Cui et al., 2010). For CA4P, although no study in UC, Lin et al. found that CA4-induced damage of vascular endothelial cells may cause demargination of white blood cells or indirectly increase white blood cells as a result of release of inflammatory factors into the plasma, suggesting CA4/CA4P possesses pro-inflammation activity in patients with refractory solid tumors (He et al., 2011). Our results show that CA4P aggravates DSS-induced colitis in mice via promoting inflammatory cell levels and infiltration, as well as release of proinflammatory cytokines.

It should be noted that the impact of CA4P on peripheral immune cells may be different between normal and disease condition, at least, at low dose. For example, in our study, CA4P alone has little impact on peripheral white blood cell counts in normal mice. While Bohn et al., observed that, CA4P mediated a uniform oscillating change in peripheral monocytes and neutrophils in tumor-bearing mice. Specifically, the effects of CA4P on cell counts during the first 6 h were similar between non-tumor-bearing and tumor-bearing mice, but the increase from 6 h was only seen in the tumor-bearing mice (Bohn et al., 2014; Bohn et al., 2015). In particular, the increase of neutrophils may attenuate CA4P-mediated anti-tumor effects when CA4P is used at high doses in some tumor types, such as C3H mammary carcinomas, suggesting that tumor associated neutrophils may diminish the effect of CA4P (Bohn et al., 2014). Indeed, the direct clinical benefits of CA4P alone as an anti-cancer agent are limited according to some clinical trial results, these initial data indicate the promise of CA4P to stimulate the immune system when in combination with other immunoregulators. In this case, Badn et al., demonstrated that a combination of low-dose CA4P and tumor immunization [like IL-18/Interferon-γ (IFNγ)] significantly enhances retardation of tumor growth as compared with either treatment alone and results in an enhanced antitumor immune reactivity (Badn et al., 2006).

Furthermore, the mechanism of CA4P-induced damage may involve antiangiogenic activity, which may create a disordered microenvironment in the colon mucosa, increased vascular resistance, and induced hemorrhage and coagulation that increased inflammation. For example, similar to the antitumor effect of CA4P, bevacizumab, a humanized monoclonal antibody against vascular endothelial growth factor, inhibits angiogenesis in NSCLC patients. However, clinical studies reported that the antiangiogenic activity of bevacizumab may have been involved in the development and exacerbation of UC in one patient (Tanaka et al., 2015). Gastrointestinal perforation is a well-described severe complication of bevacizumab treatment in patients with colorectal cancer (Hapani et al., 2009).

Taken together, this study demonstrate that CA4P does not alleviate UC, but it intensifies the occurrence of inflammatory reactions and worsens the symptoms of UC. As a clinical drug for vascular interfering agents, CA4P has been clinically effective for human cancer treatment. However, based on the effect of CA4P on UC, clinicians should consider the presence of UC or the potential of developing UC when using CA4P as a therapeutic agent to avoid triggering complications.

## Data Availability Statement

The raw data supporting the conclusions of this manuscript will be made available by the authors, without undue reservation, to any qualified researcher.

## Ethics Statement

All experimental animal procedures were reviewed and approved by the Ethics Committee of Center for Scientific Research with Animal Models of Central South University, Hunan, China. All applicable international, national, and institutional guidelines for the care and use of animals were followed.

## Author Contributions

ZW, LZ, XX, MY, and JL conceived of the study. ZT, DX, JS, and ZW carried out the experiments and performed the statistical analysis. ZW, ZT, and XX participated in its coordination and drafted the manuscript.

## Funding

This work was supported by grants from the National Natural Science Foundation of China (Nos. 81171950, 81272220, 81402304, 81672760, 81702722, and 81970195), National Postdoctoral Program for Innovative Talents (Nos. BX201700292), the Hunan Provincial Natural Science Foundation of China (Nos. 2016JJ3048 and 2018JJ3703) and the Hunan Provincial Key Research and Development Plan (Nos. 2018SK2128).

## Conflict of Interest

The authors declare that the research was conducted in the absence of any commercial or financial relationships that could be construed as a potential conflict of interest.
